# Non-equilibrium atmospheric pressure plasma assisted degradation of the pharmaceutical drug valsartan: influence of catalyst and degradation environment

**DOI:** 10.1039/d0ra05608a

**Published:** 2020-09-29

**Authors:** A. Raji, K. Navaneetha Pandiyaraj, D. Vasu, M. C. Ramkumar, R. R. Deshmukh, V. Kandavelu

**Affiliations:** Department of Chemistry, Sri Shakthi Institute of Engineering and Technology Coimbatore 641062 India; Research Division of Plasma Processing (RDPP), Department of Physics, Sri Shakthi Institute of Engineering and Technology Coimbatore 641062 India dr.knpr@gmail.com +91-9786452504; Department of Physics, School of Basic Sciences, Vels Institute of Science, Technology and Advanced Studies Chennai India; Department of Physics, Institute of Chemical Technology Matunga Mumbai 400019 India

## Abstract

This paper investigated the degradation of the pharmaceutical drug Valsartan (VS) using non-equilibrium atmospheric pressure plasma (NEAPP) with various operating conditions. The heterogeneous photocatalyst ZnO nanoparticles (NP's) were synthesized using a hydrothermal process. The morphology, chemical composition and structure of as-synthesized ZnO NPs were examined by Field Emission Scanning Electron Microscopy (FE-SEM), Fourier Transform Infrared Spectroscopy (FTIR) and X-ray diffraction (XRD) analysis. Then, VS degradation was examined in three subsequent treatment conditions including plasma treatment alone, the combination of plasma with as-prepared ZnO NPs and various environments (air, oxygen and hydrogen peroxide) at fixed plasma operating potential and treatment time. The degradation efficiency of plasma-treated VS by various conditions was observed using UV-visible spectroscopy. Optical Emission Spectrometry (OES) was used to characterize the distribution and emission intensity of various reactive species (OH˙, N_2_-SPS and O) during the degradation processes which plays a vital role in the degradation of VS. The role of OH˙ and H_2_O_2_ during the degradation process was further examined by chemical dosimetry and spectroscopic techniques. Furthermore, pH, conductivity and TOC of the untreated and plasma-treated VS were also investigated. The results on the degradation of VS showed that plasma treatment combined with ZnO NP's has a significant effect on degradation of molecules of VS than degradation processes carried out by other experimental conditions due to the formation of higher concentrations of various reactive oxygen and nitrogen species during the degradation processes.

## Abstract 2

Degradation of valsartan by plasma combined with various environmental conditions.

## Graphical abstract

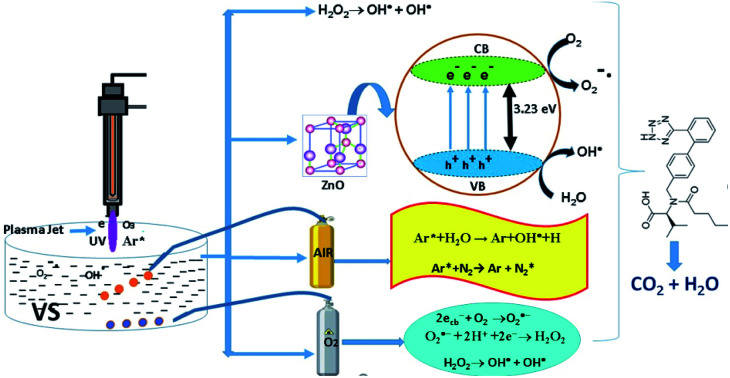

## 1 Introduction

For the past few decades, industrialization and increase in population has been adversely affecting the human health and environment. The WHO and other health organizations reported that population growth and pollution are the main causes for the increasing number of human diseases worldwide.^1^ Hence, the pharmaceutical industry plays a significant role in developing medication for reducing the chronic diseases. The drugs like carteolol, amlodipine, metformin, aspirin, terbutaline and mitomycin are common to treat the diseases like heart disease, hypertension (high blood pressure), diabetes, stroke, asthma and cancer respectively. Valsartan (VS) (3-methyl-2-[pentanoyl-[[4-[2-(2*H*-tetrazol-5-yl)phenyl]phenyl]methyl]amino]-butanoic acid) is an Angiotensin II receptor blockers (ARBs),^2^ used to treat hypertension, cardiovascular diseases and diabetic kidney diseases.^3^ According to WHO report (2013), more than 1.5 billion people suffer from cardiovascular diseases^4^ and hence worldwide consumption of valsartan is significantly increased. Prolonged consumption of this drug is reported to produce a severe allergic reaction, difficulty in breathing, depression and vision problems. On the other hand, valsartan given/prescribed for the treatment of hypertension, is not completely metabolised (>96% excreted unchanged)^5^ and eliminated as a biologically active compound to the environment which adversely affects aquatic life.^6^ Recent studies about waste water (WW)/sewage treatment plant (STP) reported that, valsartan is one of the most existing contaminations compared with other commonly used drugs.^7^ For instance, around 6.0 μg L^−1^ of VS in WW/STP,^8^ 19.8 μg L^−1^ VS in hospital effluent^7^ and 0.09 ng L^−1^ VS in marine and estuarine water (San Francisco Bay, in the USA) has found. Even though, the presence of pharmaceutical compounds in very few extent in the range of nanogram level, it can produce acute and chronic effects on human health and aquatic life.^9^

For these reasons, various attempts have been made to remove pharmaceuticals compounds from waste water. Various conventional methods such as adsorption, coagulation and filtration has been involved to degrade the pharmaceutical compound containing waste water. However, Conventional treatment method cannot completely degrade the pharmaceutical compounds^10,11^ and it generates the secondary pollutants. Advanced Oxidation Process (AOP's) is one of the very active methods to overpower the limitation of conventional treatment methods.^12–14^ In AOP, various reactive oxygen and nitrogen species (ROS and RNS) such as OH˙, O, NO^2−^, NO^3−^, and H_2_O_2_ during the degradation process which powerfully attack the toxic organic pollutants and convert them into nontoxic substance such as CO_2_ and H_2_O.^15,16^ Nevertheless, in many cases, instead of complete oxidation, it produce partial oxidation products and it again generate secondary pollutants to the environment.

Recently, non-equilibrium atmospheric pressure plasma (NEAPP) have great potential of complete oxidation of pharmaceutical compounds present in waste water *via* the production of various primary 
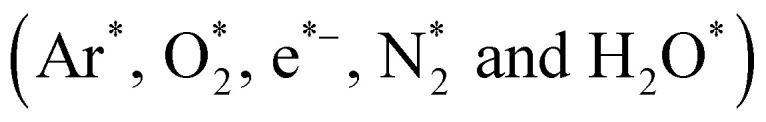
 and secondary reactive species (OH˙, O, O_3_ and H_2_O_2_) at gas phase and gas–water interface. These primary and secondary reactive species can efficiently degrade the organic pollutants present in the waste water.^17,18^ Jean Marie Herrmann^19^ reported that, heterogeneous photocatalyst (TiO_2_), totally degrade and mineralize large variety of organic and inorganic aqueous pollutant into CO_2_ and harmless inorganic anions.

Hence, we attempt to degrade the VS compounds *via* NEAPP at various operating conditions such as Ar plasma, the combination of Ar plasma with ZnO NP's and various environments (air, oxygen and hydrogen peroxide). The choice of ZnO is on the basis of its cost effectiveness, thermal stability at room temperature, high electron mobility, high photochemical reactivity, stability to photo-corrosion and low toxicity than other catalyst.^20,21^ Furthermore, it was synthesized by hydro-thermal method and was characterized by various techniques such as Field-Emission Scanning Electron Microscopy (FE-SEM), Fourier transform infrared spectroscopy (FTIR) and X-ray diffraction (XRD) studies. The influence of various operating conditions on degradation of VS aqueous solution was examined comprehensively. Then, VS degradation was examined in three subsequent treatment conditions including plasma treatment alone, the combination of plasma with as-prepared ZnO NP's and various environments (air, oxygen and hydrogen peroxide) at fixed plasma operating potential and treatment time.

## 2 Experimental procedure

### 2.1 Materials and methods

Zinc acetate dihydrate (Zn(CH_3_COO)_2_·2H_2_O) was procured from Sigma-Aldrich, India. The commercially available Valsartan (VS) compound obtained from Dr Reddy's lab. Analytical reagent grade methanol (CH_3_OH), hydrochloric acids (HCl) and sodium hydroxide (NaOH) were purchased from Merk, India.

### 2.2 Synthesis and characterization of ZnO nanoparticles

For the synthesis of ZnO NP's, 0.1 M of zinc acetate solution was prepared by adding 1.1 g of zinc acetate in 50 ml of methanol under continuous stirring. Subsequently, 0.2 g of NaOH was dissolved in 25 ml of methanol, the mixed solution was added drop wise into the precursor solution to maintain the pH between 8 to 11. The obtained solution was further transferred into teflon-lined sealed autoclave and heated at 120 °C for 24 hours under autogenous pressure. It was then allowed to cool naturally to room temperature. After the reaction was complete, the resulting white solid product was washed with methanol at several times, filtered and then dried in a hot air oven at 60 °C. Furthermore, the dried white precipitate was calcinated at 600 °C for 3 hours.^22^

The morphology of synthesized ZnO NP's were examined by FE-SEM (ZEISS SIGMA FESEM, Germany). The chemical composition of the ZnO NP's was analysed by FT-IR (FT/IR-4700 typeA). After the degradation process the ZnO NP's was further filtered by Whatman-40 filter paper and washed several times with DI water followed by ethanol. The obtained ZnO NP's was preheated at 60 °C and calcined at 600 °C for 3 h. Thereafter, the chemical compositions of the recycled ZnO NP's were further confirmed by FTIR. The phase and crystallinity was observed by X-ray Diffraction (XRD) (X'pert PRO X-ray Diffractometer, PANalytical, Netherland) with Cu-Kα (*λ* = 1.5406 Å and 2*θ* range from (10–90°) radiation.

### 2.3 NEAPP reactor

**Fig. 1:**
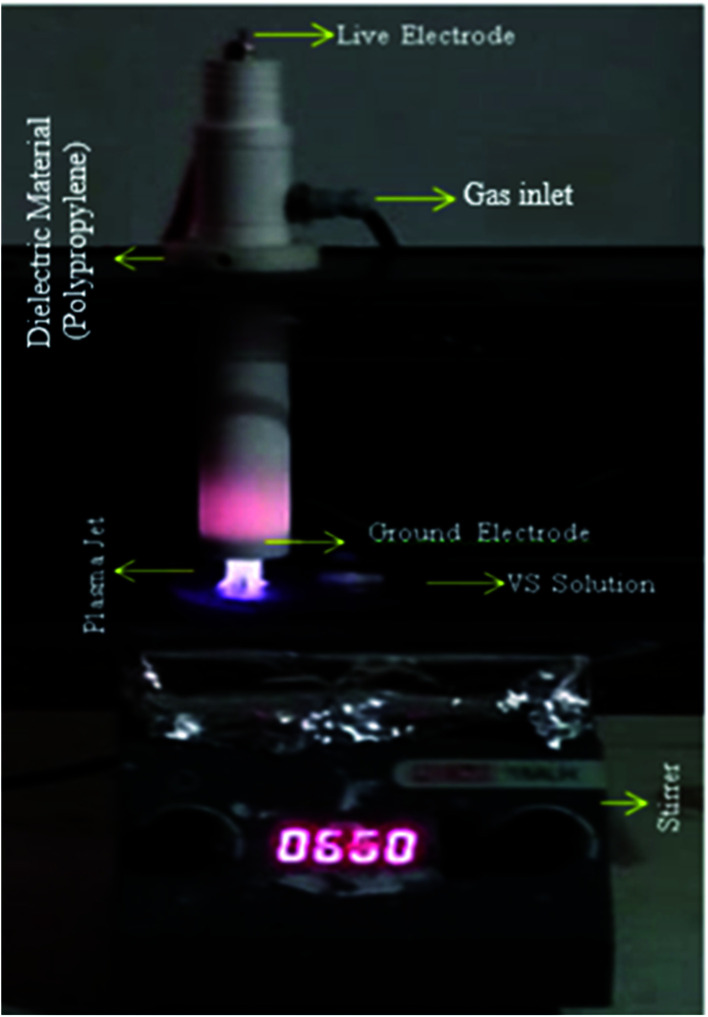
NEAPP system for VS treatment.

The NEAPP effluent treatment reactor was used for this treatment is shown in Fig. 1. The details of the plasma reactor are explained elsewhere.^23^ The major components of the system are plasma torch and high frequency and high voltage power supply. The plasma torch consists of rod-type live and ring shape ground electrode which made of copper. The live electrode is encapsulated by quartz tube in order to avoid arcing and was connected to an AC power supply which is operated with maximum voltage and frequency of 40 kV and 50 kHz. The ground ring electrode was situated just below the live electrode and the distance between them was 2.5 cm. The electrode assembly was further covered by a Teflon jacket in order to prevent electric induction during the experiment (Fig. 1). The system has a separate provision for gas inlet which is controlled by mass flow controller (AALBORG GFC37).

### 2.4 Preparation of Valsartan solution and degradation process

Initially, the stock solution having 10^−4^ mole concentration of VS was prepared for degradation process. In the first treatment condition, VS aqueous solution was poured into reaction flask and was positioned 5 mm distance below the nozzle orifice. Subsequently, argon plasma forming gas was allowed between the electrodes and high voltage was applied till the homogeneous jet was expelled *via* nozzle exit. The plasma plume was discharged from the nozzle exit and was allowed to pass into VS aqueous solution and treatment was carried out at optimized operating potential and treatment time of 35 kV and 10 min. In the second treatment condition, air was allowed into the reaction flask which leads to produce air bubbles in the aqueous solution and the same was stirred continuously using magnetic stirrer. Subsequently, the air bubbles containing aqueous solution was treated by Ar plasma at the operating parameters which was optimized for plasma treatment. Similarly VS solution was treated using O_2_ bubbles and also by adding H_2_O_2_ (1.6 × 10^−5^ M). In the final condition of degradation processes, 20 mg of ZnO NP's was dispersed into the aqueous solution containing reaction flask and consequently ZnO containing VS solution was further treated by Ar plasma. The typical parameters of plasma assisted VS aqueous solution treatment are given in Table 1.

**Table 1:** Typical parameters of plasma assisted VS aqueous solution treatment

Applied potential	35 kV
Treatment time	10 min
Distance between live and ground electrode	2.5 cm
Distance between plasma torch and water surface	5 mm
Plasma-forming gas	Argon
Ar gas flow rate	9 lpm

### 2.5 Characterization

#### 2.5.1 Identification of reactive species

The information of the reactive species produced in plasma jet during the degradation of VS was examined by an optical emission spectroscopy (OES) (Ocean Optics, HR 4000CG UV-NIR, 1 nm). The emission spectra were recorded in the wavelength region of 200–1100 nm. An optical fiber cable (QP400-2-SRBX) was used to collect the optical signals and was connected to the collimating lens in order to confine the field of view, increase the collection efficiency and the spatial resolution of spectrometer. The optical fiber setup was placed directly near the plasma aqueous solution treatment region using glass feed through. The spectral measurement was further diagnosed by Ocean view software.

#### 2.5.2 Detection of OH radicals and H_2_O_2_

Terephthalic dosimetry is one of the chemical methods to quantify the OH radicals produced during the degradation process. Terephthalic Acid (TA) is an excellent OH radical scavenger at the pH range of 10–11. TA does not react with other reactive species such as H_2_O_2_, O^2−^*etc.* TA molecule is reacted with OH radical to form 2-hydroxy terephthalic acid (HTA) which can be detected by fluorescent method. UV light (*λ* = 310) is irradiated in the aqueous solution containing TA and HTA. The HTA molecule alone emits the radiation at *λ* = 425 nm in UV region, whereas TA molecule does not emit. The concentration of HTA molecules are proportional to the concentration of OH radicals which directly related with the intensity of fluorescence emission.^24,25^

Potassium titanium(iv) oxalate (K_2_TiO (C_2_O_4_) 2H_2_O) method is used to determine the concentration of H_2_O_2_ by spectrophotometric method. Initially, 3.5 g of K_2_TiO (C_2_O_4_) 2H_2_O was added to mixed solution of concentrated H_2_SO_4_ (27.2 ml) and deionised water (30 ml). The titanium reagent solution was further made up to 100 ml by using DI water. For analysis, 5 ml of titanium reagent and 5 ml of VS solution were taken into a calibrated flask and made up to 25 ml by adding DI water. The solution was treated by above mentioned operating conditions and the production of H_2_O_2_ was measured using spectrophotometer at a wavelength of 420 nm.^26,27^

The concentration of H_2_O_2_ (mol L^−1^) was calculated as follow1
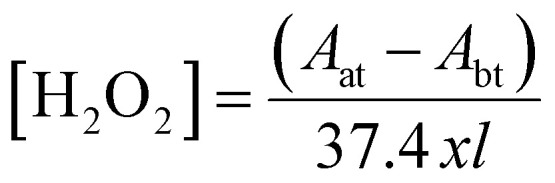
where *A*_bt_ and *A*_at_ are the absorbance (at *λ*_max_) of the untreated and plasma treated solutions respectively. *x* and *l* are the volume of the solution and the path length (cm) of the spectrophotometer cuvette respectively.

#### 2.5.3 Investigation of degradation processes

The degradation efficiency of plasma treated VS aqueous solution with different treatment condition was observed using UV-Vis spectrophotometer (OCEAN Optics HR4000) equipped with Deuterium Halogen light source. The % of degradation of VS aqueous solution due to plasma treatment was calculated by the following formula.^28^2
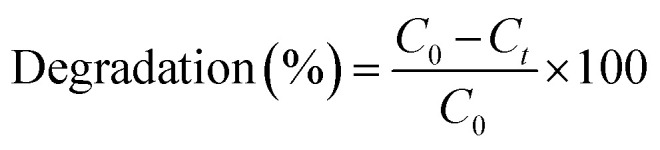
where, *C*_o_ and *C*_*t*_ are the initial and final (after plasma) concentration of VS solution. The conductivity and the pH of the degraded VS solution were measured using a digital electrical conductivity meter-611 (Elico Ltd, India) and a digital pH meter (pHep, HANNA Instruments, USA) respectively. Total organic carbon analyzer (Shimadzu TOC-LPH) was used to determine the percentage of removal of carbon from the degraded aqueous VS solution. The percentage of TOC removal of VS aqueous solution was calculated using the following relation.^29,30^3
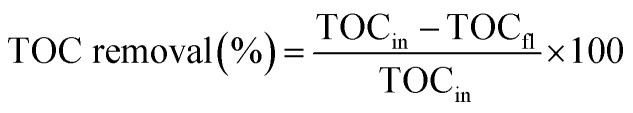
where, TOC_in_ and TOC_fl_ are the initial and final concentrations of TOC in VS aqueous solution respectively.

## 3 Results and discussion

### 3.1 Catalysis characterization

**Fig. 2:**
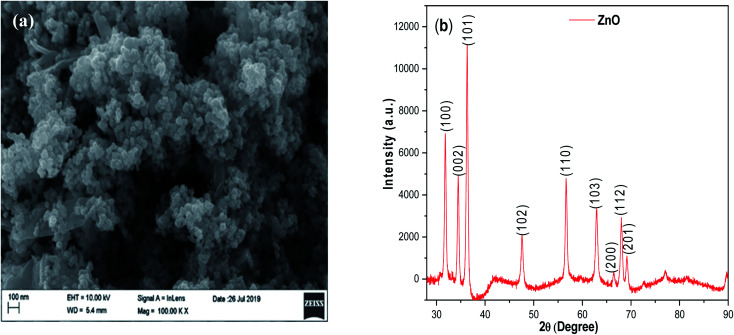
(a) SEM image and (b) XRD pattern of synthesized ZnO nano particles.

The morphology of synthesized ZnO NP's was observed by FE-SEM which exhibits that the morphology of ZnO NP's has crystalline structure comprising of aggregated spherical particles (Fig. 2a). Fig. 2b shows the XRD pattern of synthesized ZnO NP's, it is clear that the synthesized ZnO NP exhibit crystalline structure with sharp peaks located at 2*θ* = 31.61, 34.61, 36.40, 47.52, 56.67, 62.73, 66.49, 67.80 and 69.10 were assigned to (100), (002), (101), (102), (110), (103), (200), (112) and (201) respectively, resembles the wurtzite hexagonal phase of ZnO. The observed powder pattern was compared with the standard JCPDS card (36-1451). The average crystallite size of the synthesized ZnO Np catalyst was ∼20 nm calculated using the Debye-Scherer's formula *D* = (0.94*λ*)/(*β* cos *θ*), (where *λ* is the wavelength of X-ray (Cu-Kα radiation), *β* is the full width at half maximum intensity and *θ* is the Bragg's angle.

### 3.2 FTIR result

**Fig. 3:**
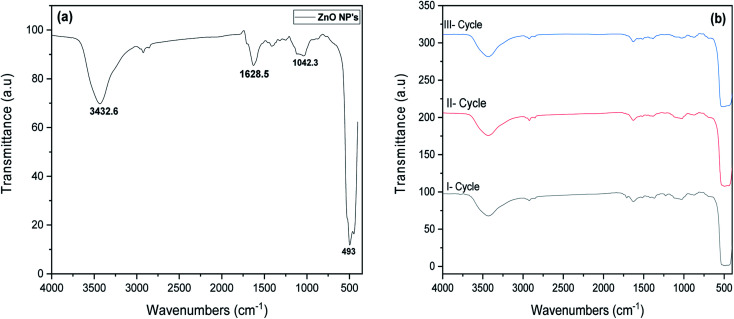
FTIR spectra of (a) synthesised ZnO NP's and (b) ZnO NP's for various recycles.

The chemical composition of ZnO NP's was studied by FTIR spectra as displayed in Fig. 3a. It exhibits major peaks corresponding to stretching vibration mode of ZnO bond around 493 cm^−1^. The presence of peaks at 3432.6 cm^−1^ and 1628.6 cm^−1^ attributed to O–H stretching vibration and the adsorption of water on the particles surface.^31,32^ FTIR results confirm that the major dominance of functionalities in the NP's was ZnO bond. Fig. 3b, depicted the FTIR spectrum of recycled ZnO NP's after degradation processes. It implies that after three cycles, no significant change was observed in the chemical composition of ZnO NP's which confirms the retention of functional groups before and after recycling process.

### 3.3 Determination of reactive species present in various environments: OES analysis

**Fig. 4:**
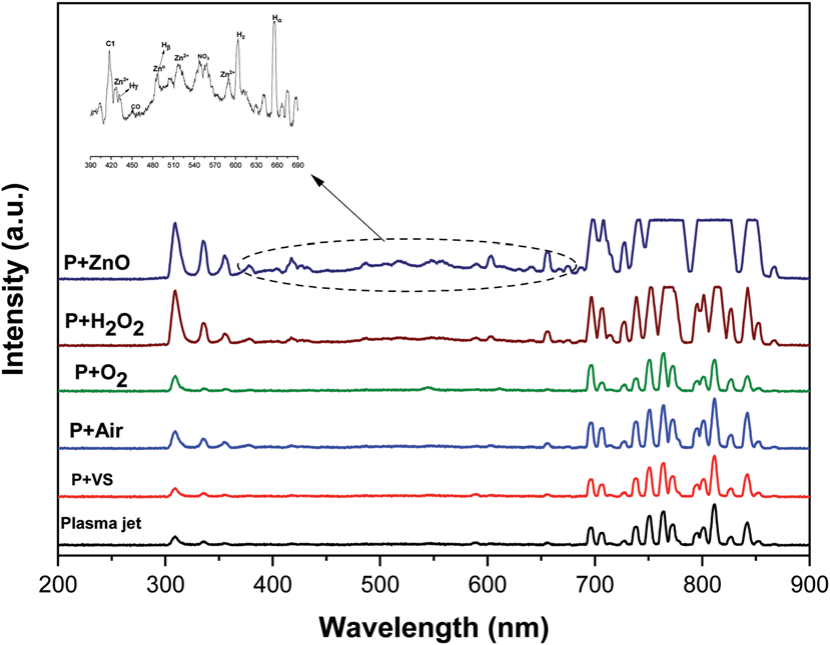
Optical emission spectrum of NEAPP jet with various operating conditions.

Fig. 4 implies the OES spectra of argon plasma jet during degradation of VS for various operating conditions. It was observed that OES spectra of Ar plasma jet (before degradation) exhibits various spectral lines due to argon excited species (Ar*) (4s ← 4p lines) (690–900 nm), OH radicals (309 nm), atomic oxygen (3s^5^S ← 3p^5^P) (772 and 842 nm)^33,34^ and nitrogen second positive system (N_2_–SPS) (B^3^Π_g_ ← C^3^Σ_u_) (336, 354 and 380 nm). The formation of identified reactive species such as OH˙, N_2_-SPS and O (reactive nitrogen species (RNS) and reactive oxygen species (ROS)) in plasma jet may be attributed to the diffusion of ambient gas molecule and moisture in the atmosphere into the plasma jet causes the formation of ROS and RNS *via* following reaction mechanism^35,36^

R1 Ar* + H_2_O → Ar + OH˙ +H

R2 e^−^* + O_2_ → 2O˙ + e^−^

R3
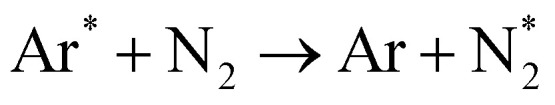
R4
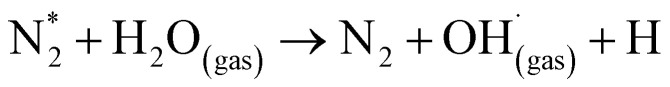


The OES spectrum of Ar plasma jet during degradation of VS aqueous solution exhibits two new peaks due to H line of the Balmer series such as Hα (at 655.35 nm) and Hβ (at 588.31 nm). The formation of Hα and Hβ may be attributed to dissociation of water molecule (H_2_O + e^−^→ OH˙+ H^+^+ 2e^−^) during the processes. However, the intensities of spectral lines due to OH˙, O and N_2_-SPS were found to be increased slightly when compared with that of spectral lines observed in Ar plasma alone. A similar spectral lines was observed when degradation of VS aqueous solution carried out by the Ar plasma at various environment such as air, O_2_ and H_2_O_2_. However, the intensity of spectral lines found to be increased in the order of P + air < P + O_2_ < P + H_2_O_2_ due to the formation of higher concentration of ROS and RNS during the degradation processes with respect to the environment of degradation. When Ar plasma degradation processes combined with ZnO NP's the OES exhibits various new spectral lines at 483, 434, 517 and 590 nm corresponds to Zn and Zn^2+^.^37^ The formation of species may be due to the interaction of plasma species with ZnO NP's cause's excitation of Zn during the degradation of VS which plays a significant catalytic role during degradation processes and stimulates oxidation reactions. Furthermore, the intensity of spectral line due to OH˙, O and N_2_-SPS were increased substantially compared with the degradation processes carried out at various environment due to the formation of higher concentration of reactive species than the other treatment condition which will be discussed more detail in section 3.4.

### 3.4 Investigation of plasma treated VS solution with different environment-UV analysis

**Fig. 5:**
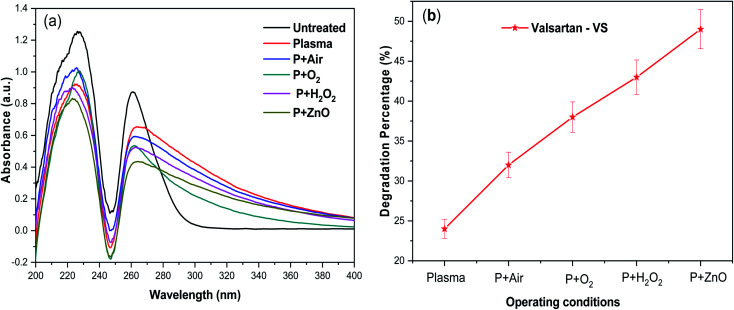
(a) UV-vis spectra of the plasma-treated VS drug and (b) degradation percentage in presence of various operating conditions.

Fig. 5a, shows the UV-vis absorbance spectra of VS aqueous solution degraded as a function of various treatment conditions. The UV-vis spectrum corresponding to untreated VS aqueous solution exhibited major absorbance characteristic peak at 260 nm. After, plasma treatment alone, the intensity of the absorbance peak was found be decreased significantly which indicating that 24% of the VS molecules was decomposed during the processes (Fig. 5b). After plasma degradation carried out at various environment, the intensity of the absorption peak decreased in the order of P > P + air > P + O_2_ > P + H_2_O_2_ which indicates an increase in the degradation percentage of VS aqueous solution. The above changes may be correlated to the oxidative degradation of VS molecules in aqueous solution due to the formation of primary 
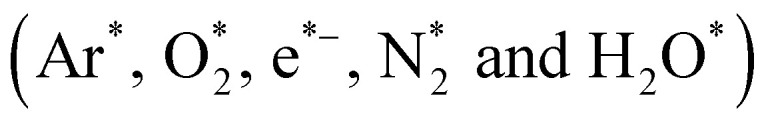
 and secondary reactive species (OH^.^, O, O_3_ and H_2_O_2_) during the plasma degradation processes. Finally, the plasma treatment combined with ZnO NP's, obtained lower intense of absorbance peak with maximum degradation percentage of 49% compared to other treatment conditions which mainly caused by the formation of higher concentration of various reactive species during this synergetic process by following facts. Interaction of electron and excited species in plasma with molecules of aqueous solution leads to produce various ROS 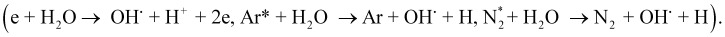 In spite of reactive species, high intense of UV photon in plasma has also contributed to produce various reactive species by photocatalysis mechanism. The ZnO NP's in aqueous solution absorbs UV photons and forms an electron and hole pairs (e^−^_CB_ + h^+^_VB_). This photo induced electron–hole pair migrate to the surface of ZnO undergoes redox reaction to produce large number of OH˙ radicals, super oxide anion radicals (O^2^˙^−^) and hydroperoxy radicals (HO_2_˙) *via* following reaction.

R5 ZnO + *hγ*→ ZnO (e^−^_CB_ + h^+^_VB_)

R6 ZnO(h^+^_(VB)_) + H_2_O → ZnO + H^+^ + OH˙

R7 ZnO(h^+^_(VB)_) + OH^−^ → ZnO + OH˙

R8 ZnO(e^−^_(CB)_) + O_2_ → ZnO + O^2^˙^−^

R9
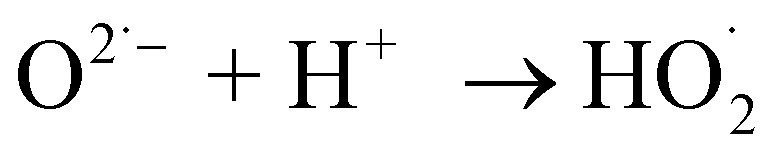
R10
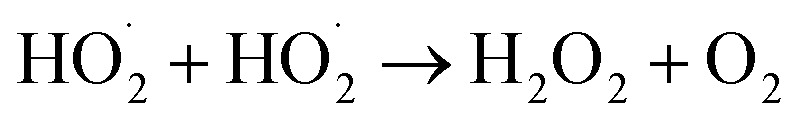

R11 ZnO(e^−^_(CB)_) + H_2_O_2_ → OH˙ + OH^−^

R12 H_2_O_2_ + O^2^˙^−^ → OH˙ + OH^−^ + O_2_

R13 H_2_O_2_ + *hv* → 2OH˙

In this way higher concentration of various reactive species are produced in the plasma degradation processes combined with ZnO NP's which are facilitated to decompose VS in the aqueous solution. Comparison of degradation of VS by this technique with other treatment methods was given in Table 2. It is evident that combination of NEAPP with ZnO NP's yield higher degradation efficiency within shorter treatment time compared with other AOP due to the formation of higher concentration of reactive species.

**Table 2:** Comparison of degradation of VS by this technique with other treatment methods

Sl/no	Treatment method	Catalyst	Treatment time (min)	% of degradation efficiency	Ref.
1	AOP	Photo-Electro-Fenton	90	70%	38
2	AOP	Ti/IrO_2_ doped with SnO_2_	120	100%	39
3	AOP	Sona chemical	30	60%	40
4	AOP	Electro chemical	180	100%	41
5	AOP	NEAPP + ZnO	10	49%	This work

### 3.5 Detection of OH˙ and H_2_O_2_ spectroscopy analysis

**Fig. 6:**
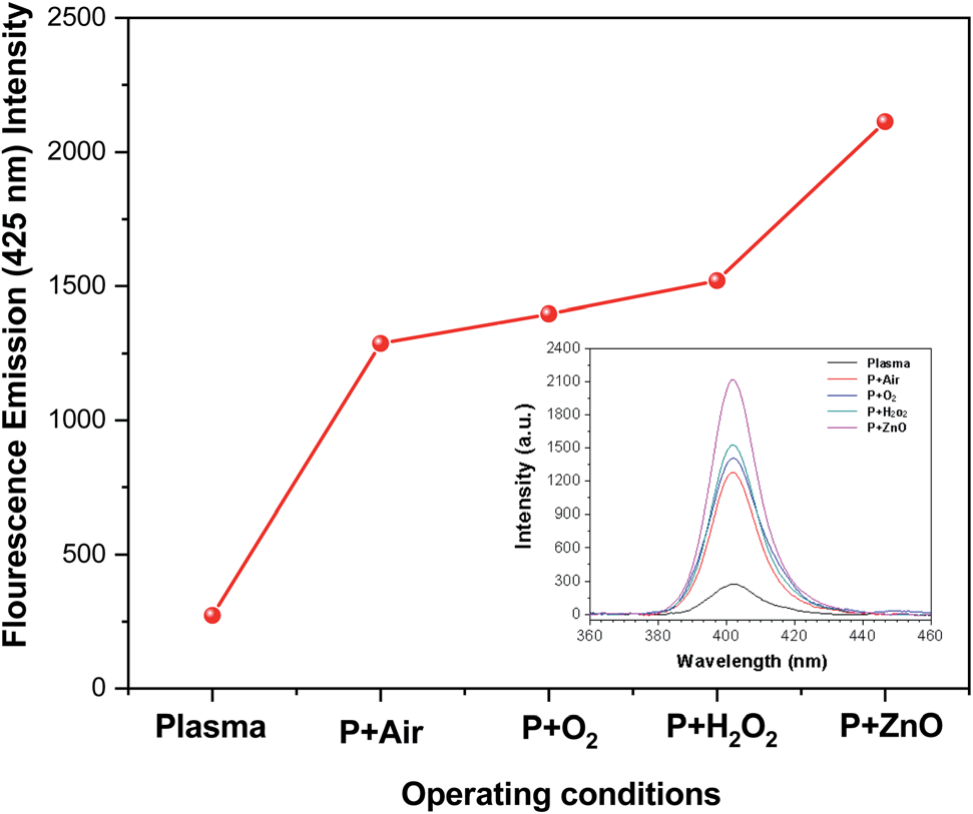
Concentration of OH radicals in plasma-treated VS solution with various operating conditions.

In radical chemistry and radiation chemistry OH radical plays a vital role due to their high oxidative potential (2.8 V). The quantity of OH˙ radicals during the plasma process was analysed by UV-Visible spectroscopy. The UV light can be easily detected with terephthalate, which react with OH˙ radicals to form hydroxyl terephthalic acid (fluorescent product) which has fluorescent emission at 425 nm. The intensity of fluorescent emission is proportional to the production of OH˙ radicals. The amount of OH radicals formed during the degradation of VS aqueous solution under different environment such as plasma, air, oxygen, H_2_O_2_ and ZnO Np's (at constant applied potential of 35 kV and treatment time of 10 min) is reported in Fig. 6. In plasma, the excited Ar reacts with aqueous solution and H_2_O_2_ to produce OH radicals which exhibits the fluorescence emission intensity of 263. During the degradation of VS aqueous solution with different environment, the production of OH radicals considerably increased and the fluorescence emission intensity follows the order: plasma < P + air < P + O_2_ < P + H_2_O_2_ < P + ZnO at 35 kV and 10 minutes of treatment time. Comparing with various treatment conditions, ZnO NP's shows highest fluorescence intensity of 2123 due to the higher production of OH radicals during the reaction.

**Fig. 7:**
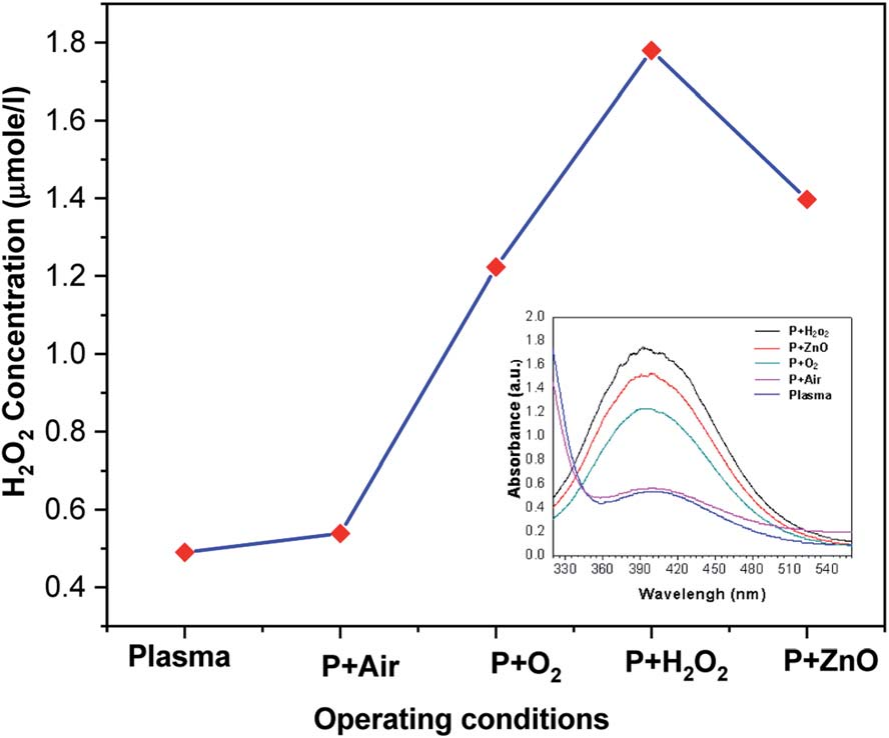
Concentration of H_2_O_2_ in plasma-treated VS solution with various operating conditions.

Amount of H_2_O_2_ formed during the degradation of VS in presence of various treatment conditions is depicted in Fig. 7. Generally; high energy electron dissociates the water molecule to produce H_2_O_2._ It is clear from Fig. 7 that the *in situ* production and external addition of H_2_O_2_ increases the amount of hydrogen per oxide during the reaction. But the concentration of H_2_O_2_ is lower in the case of ZnO catalyst. The presence of ZnO catalyst, photo induced electrons and holes dissociated the H_2_O_2_ into OH radicals. Hence, compared with other condition (H_2_O_2_), the amount of H_2_O_2_ was found to be less in the case of ZnO NP's.

The production of H_2_O_2_ during the reaction is given below

R14 OH˙ + OH˙ → H_2_O_2_

R15 O + H_2_O → H_2_O_2_

R16 OH˙ + OH˙ + Ar˙→ H_2_O_2_ + Ar

R17 OH˙ + HO_2_ +Ar˙ → H_2_O_2_ + O + Ar

### 3.6 Variation of pH, electrical conductivity and TOC

**Fig. 8:**
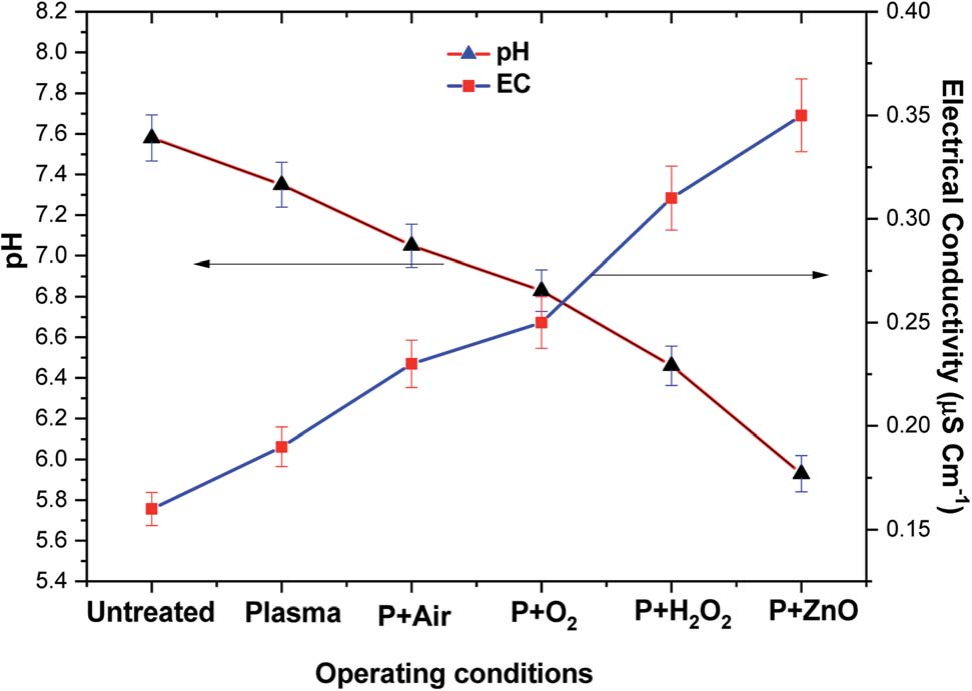
Electrical conductivity and pH of VS aqueous solution as a function of various operating conditions.

Fig. 8 depicts the changes in pH and EC of VS aqueous solution with respect to various treatment conditions. It was observed that the pH and EC of untreated VS aqueous solution was 7.6 and 0.15 μS cm^−1^. After plasma treatment the pH of the VS aqueous solution was found to decreased in the order of P > P + air > P + O_2_ > P + H_2_O_2_ > P + ZnO, whereas the conductivity was increased in an opposite trend of pH. The above change may be attributed to the formation of various acid (HNO_3_ and HNO_2_) and ionic species (NO^2−^ and NO^3−^) in the aqueous solution by conversion of nitrogen containing reactive species such as N, NO and NO_2_ during the treatment by the following reaction.^42,43^

R18 N_2_ + e→ N˙ + N˙

R19 N˙ + 2O˙ → NO_2_

R20 N˙ + O_2_→ NO + O˙

R21 NO + O_3_→ NO_2_ + O_2_

R22 NO^2−^ + O_3_→ NO^3−^ + O_2_

R23 NO^2−^ + 2OH˙ → NO^3−^ + H_2_O

R24 NO_2_ + OH˙ → HNO_3_ → H^+^ + NO^3−^

R25 NO + OH˙ → HNO_2_ → H^+^ + NO^2−^

**Fig. 9:**
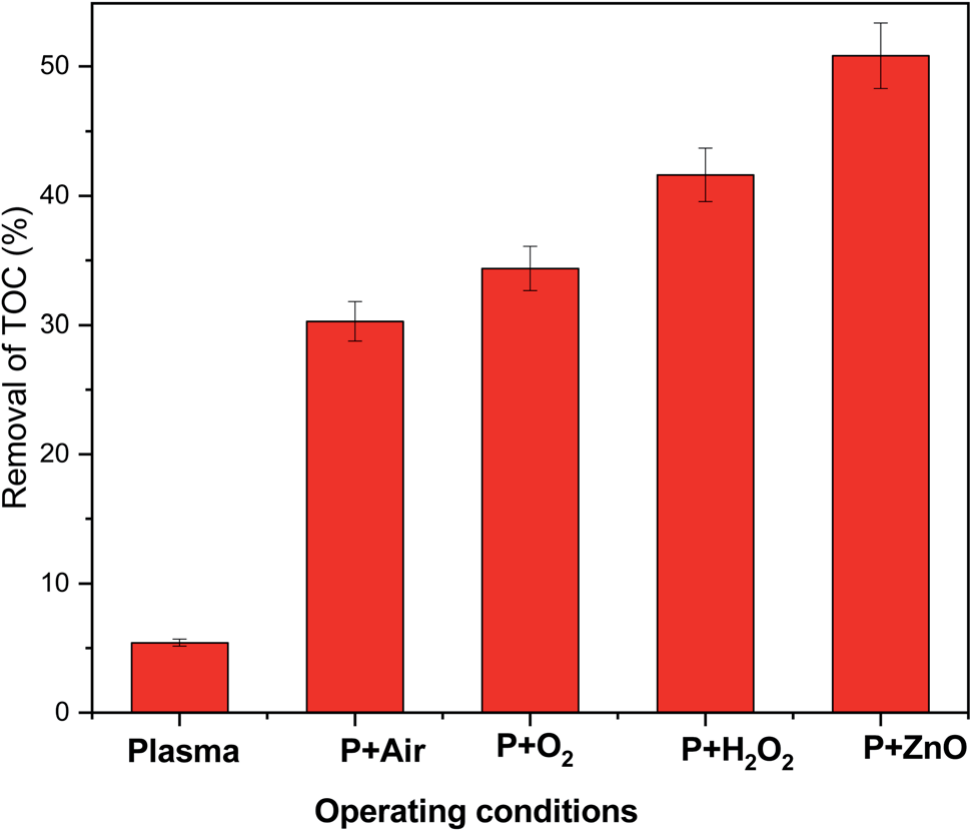
TOC removal in VS aqueous solution as a function of various operating conditions.

The mineralization of VS aqueous solution by various plasma treatment condition were examined by measuring elimination of TOC in aqueous solution (Fig. 9). It was observed that 5.43% of TOC was removed from the VS aqueous solution by Ar plasma treatment alone and was found to be increased by the degradation processes carried out by the following order P < P + air < P + O_2_ < P + H_2_O_2_ < P + ZnO, this may be due to formation of various reactive species during the processes. The obtained reactive species further dissociate/oxidized the carbon network of an organic compounds into small fragments of carbon molecules which may be further converted into various acid and finally to carbon dioxide (CO_2_). The variation in pH, electrical conductivity and TOC removal percentage clearly implies that the mineralization of VS aqueous solution was achieved by plasma treatment.^44^

## 4 Conclusion

The NEAPP in an aqueous solution containing pharmaceutical drug VS with ZnO NP's showed higher degradation (%) compared with other treatment conditions such as plasma alone, air, O_2_ and H_2_O_2_ due to the formation of higher concentration of ROS confirmed by spectroscopic analysis. Moreover, a decrease in pH, increase in conductivity and percentage of TOC removal results supports that substantial degradation of VS aqueous solution in the order of P < P + air < P + O_2_ < P + H_2_O_2_ < P + ZnO. Finally, we conclude that VS degradation carried out by the combination of plasma with catalyst exhibited significant efficacy than other treatment conditions.

## Conflicts of interest

There are no conflicts to declare.

## Supplementary Material
